# Multisystem hemorrhage in a very preterm infant born to a mother with immune thrombocytopenia: a case report and literature review

**DOI:** 10.3389/fped.2026.1803525

**Published:** 2026-06-03

**Authors:** Kunzhen Liu, Maojun Li, Wei Shi, Yanmei Wu, Qian Yang

**Affiliations:** Department of Pediatrics, Sichuan Provincial People’s Hospital, University of Electronic Science and Technology of China, Chengdu, Sichuan, China

**Keywords:** health of infants and young children, ITP, multisystem hemorrhage, neonatal immune thrombocytopenia (NITP), very preterm infant

## Abstract

**Background:**

Maternal immune thrombocytopenia (ITP) may cause neonatal ITP (NITP) via transplacental IgG. Evidence in very-preterm infants remains limited.

**Case presentation:**

A male infant born at 28 + 3 weeks (1,100 g) to a mother with long-standing ITP developed severe early-onset thrombocytopenia (nadir 26 × 10⁹/L) and multisystem hemorrhage (pulmonary, gastrointestinal, and Papile grade III intraventricular hemorrhage). Despite three courses of intravenous immunoglobulin (IVIG, 1 g/kg each) and multiple platelet (PLT) transfusions, early hematologic responses were suboptimal; PLT counts began to rise on day-of-life 14 and normalized by day 19. Bone-marrow examination showed impaired megakaryopoiesis, and no definitive pathogenic genetic variant was identified.

**Discussion:**

The presentation suggests a multifactorial pathogenesis in very-preterm NITP—maternal antibody–mediated PLT destruction, gestational-age-related limits in PLT production, and potential genetics-mediated immune dysregulation. Variable IVIG exposure/effect in preterm infants may relate to developmental FcRn biology. Mechanism-aligned care may include careful IVIG dosing/timing, judicious PLT transfusion with immunologically guided selection, and targeted immune-genetic evaluation in IVIG-suboptimal responders.

**Conclusion:**

The manuscript reports a case of a very preterm male infant born to a mother with ITP, who presented with severe thrombocytopenia and multisystem hemorrhage that was difficult to manage. This case highlights the necessity of an active and comprehensive management strategy for such infants to provide individualized interventions for the immune thrombocytopenia while concurrently addressing general problems associated with prematurity.

## 1 Introduction

Immune thrombocytopenia (ITP) is an autoimmune bleeding disorder characterized by the production of autoantibodies that target platelet (PLT) surface antigens. These pathogenic immunoglobulin (Ig)G-class anti-PLT antibodies can cross the placenta, entering the fetal circulation, and in pregnant females with ITP, this transplacental movement may lead to neonatal ITP (NITP). NITP incidence is estimated to be approximately 15% to 30%, and some affected neonates may develop serious hemorrhagic complications, including intraventricular hemorrhage (IVH) (1–3). Severe bleeding episodes not only directly threaten the life of newborns, but the resulting neurological sequelae may also exert profound impacts on long-term quality of life and neurodevelopmental outcomes in infants and young children, posing a significant challenge to early childhood health management.

Although NITP is a widely recognized immune-mediated hemorrhagic disorder in newborns, its pathophysiology, clinical manifestations, and therapeutic responses in very preterm infants (gestational age 28 to <32 weeks) remain unelucidated (4, 5). Very preterm infants with NITP may present notable susceptibility to multisystem hemorrhage, considering the inherent immaturity of their hematopoietic and immune systems, along with the structural fragility of their vasculature, posing typically challenges for clinical management (6). As the most vulnerable subgroup within the infant and young child population, the fundamental health of extremely preterm infants is highly susceptible to compromise due to complications such as NITP. Therefore, in-depth investigation into its unique disease characteristics is crucial for improving both their short-term prognosis and long-term health outcomes.

Presently, most studies focus on term or late preterm neonates with NITP, with limited studies on very preterm populations (4, 5). Furthermore, in-depth investigations are required on the interplay among immune-mediated PLT destruction, impaired thrombopoiesis, and genetic factors in this vulnerable subgroup (4–9).

Additionally, individual variability in response to immunotherapy, particularly in cases with delayed or inadequate efficacy, remains underexplored (4, 5, 10–12). This report presents a case of a very preterm male infant who was born at 28 weeks and 3 days of gestation to a mother with a history of ITP. The neonate presented with severe thrombocytopenia immediately post-birth, followed by pulmonary hemorrhage, gastrointestinal bleeding, and grade III IVH. Moreover, the infant exhibited delayed therapeutic response despite receiving multiple courses of intravenous Ig (IVIG). Subsequently, bone marrow analysis and whole-exome sequencing (WES) revealed abnormal megakaryopoiesis, and whole exome sequencing did not identify any definitive pathogenic variant.

The findings of this case highlight that antibody-mediated PLT destruction is not the sole factor contributing to NITP, especially in extremely premature infants. Its development may involve a multifactorial pathogenesis, including impaired PLT production (5, 13, 14) and dysregulated clearance pathways—FcRn-regulated IgG catabolism and antibody-mediated PLT clearance (10, 15, 16). The delayed response to IVIG suggests that potential genetic factors may influence treatment efficacy. In addition, the baseline immune immaturity of very preterm infants may compound impaired immunologic platelet recovery (8). The detailed clinical course and diagnostic workup of the reported case, along with a literature review, contribute to the characterization of the distinctive clinical features, underlying pathophysiology, and individualized treatment considerations of NITP in extremely preterm infants. Through this case report, this study aims to uncover the complex factors influencing the occurrence and therapeutic response of neonatal immune thrombocytopenia (NITP) in extremely preterm infants, emphasizing the necessity of implementing multisystem and multidimensional assessments alongside individualized interventions from a holistic “infant and child health” perspective. The findings of this study may offer novel insights and a potential conceptual framework for advancing precision diagnosis and management in this vulnerable population, ultimately improving health outcomes during infancy and early childhood.

## 2 Case description

The patient was a male neonate born at 28 weeks and 3 days of gestation via cesarean section (birth weight was 1,100 g). The mother had received antenatal dexamethasone for fetal lung maturation. The Apgar scores were 7 and 9 at 1 and 5 min, respectively. The maternal parent presented with a documented 10-year history of primary ITP, which was diagnosed before conception. During pregnancy, the female reached a PLT count of nearly 4 × 10⁹/L and consequently was started on intermittent treatment with recombinant human thrombopoietin (TPO, TPIAO). Third-trimester ultrasound revealed fetal growth restriction, and antenatal assessment indicated a high risk of preterm delivery.

### 2.1 Presentation on admission and early complications

The infant was admitted due to “poor responsiveness and respiratory distress after birth.” Physical examination on admission revealed lethargy, poor responsiveness, decreased muscle tone, and absent primitive reflexes. Respiratory examination showed subcostal retractions, and moist rales were audible in both lungs, necessitating endotracheal intubation and mechanical ventilation support. The circulatory system was characterized by cool extremities and a capillary refill time of 2 s. Arterial blood gas analysis on admission indicated mild respiratory alkalosis, with an oxygenation index (P/F) of 200. Early ancillary tests: Bedside chest radiography was consistent with neonatal respiratory distress syndrome; echocardiography revealed a hemodynamically significant patent ductus arteriosus (PDA, 4 mm in diameter); and cranial ultrasonography indicated bilateral subependymal hemorrhage (Grade II IVH). Laboratory tests showed, in addition to severe thrombocytopenia (26 × 10⁹/L), the presence of leukopenia, hypofibrinogenemia, and stress hyperglycemia. Based on the clinical presentation, a diagnosis of neonatal sepsis and respiratory failure was made, and anti-infective therapy was initiated. Specific anti-infective regimen: Initially, penicillin (15,000–25,000 U/kg q12 h, intravenous infusion) plus ceftazidime (50 mg/kg q12 h, intravenous infusion) was administered. Due to the severity of the illness and high risk of drug resistance, the regimen was subsequently escalated to meropenem (20 mg/kg q8 h, intravenous infusion) combined with vancomycin (15 mg/kg q12 h, intravenous infusion), with dose adjustments based on renal function. The course of treatment was continued until infectious markers and body temperature normalized, and both blood culture and blood metagenomic next-generation sequencing (NGS) showed no abnormalities (approximately on day 22 after birth). Concurrently, pulmonary surfactant was instilled via the endotracheal tube, caffeine (loading dose 20 mg/kg, maintenance dose 5–10 mg/kg/day) was given to stimulate the respiratory center, and the ventilator mode was initiated with conventional frequency ventilation, with subsequent parameter adjustments based on oxygenation and blood gas analysis.

### 2.2 Occurrence and management of multisystem hemorrhage

On days 2-3 of life, the infant experienced sudden respiratory deterioration, with bloody secretions observed in the endotracheal tube. Combined with imaging findings, a diagnosis of pulmonary hemorrhage was made. At this stage, the infant concurrently presented with a left-to-right shunt due to PDA and a septic state. Endotracheal administration of diluted epinephrine (1:10,000 solution, 0.5–1 mL per dose); intravenous ethamsylate (10 mg/kg per dose, every 6–8 h) for hemostasis; infusion of fresh frozen plasma (10–15 mL/kg per dose); infusion of random donor platelets (10–15 mL/kg per dose). On day 8 of life, the infant developed hematemesis and coffee-ground-like material in the gastric tube, leading to a diagnosis of gastrointestinal hemorrhage. Fasting for 24–48 h was implemented, along with intravenous omeprazole at 1 mg/kg every 12 h for acid suppression, and platelet transfusion (10–15 mL/kg per dose). After bleeding was controlled, feeding was gradually resumed, starting with 2 mL of breast milk as a trial, then increased to 45 mL per dose of preterm infant formula every 3 h. During this period, serial cranial ultrasound monitoring revealed progression of IVH to Grade III accompanied by ventricular dilation (maximum width reaching 16 mm). To alleviate hydrocephalus, serial lumbar punctures were performed for cerebrospinal fluid (CSF) drainage, initially once daily or every other day, with 5–10 mL of cerebrospinal fluid removed each time, until pressure, protein, and cell counts returned to normal. CSF examination ruled out intracranial infection, and the findings were attributed to bleeding. Subsequent, a cranial MRI performed on day 34 of life (Figure 1A) showed grade III IVH with ventricular dilation; a follow-up cranial MRI on day 49 of life (Figure 1B) confirmed changes in the absorption phase of hemorrhage and ventricular enlargement, with no significant parenchymal injury observed.

**Figure 1:**
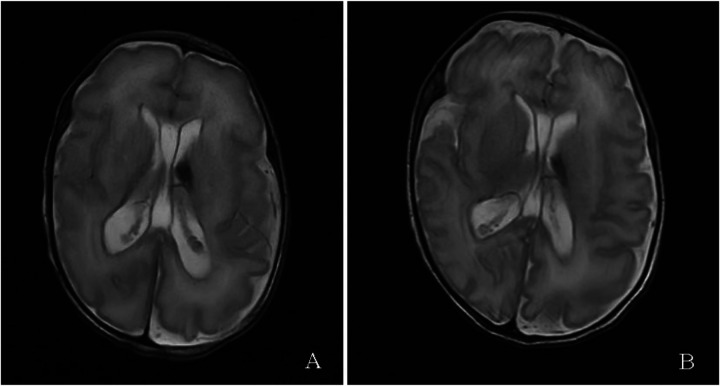
**(A)** Brain magnetic resonance imaging (MRI) showing grade III intraventricular hemorrhage (IVH) with associated ventricular dilation (day 34) sequences and slice levels are indicated where applicable. **(B)** Follow-up MRI showing substantial resolution of IVH with no evidence of parenchymal injury (day 49).

### 2.3 Investigations and management of thrombocytopenia

The infant's thrombocytopenia was attributed to maternal disease. Intravenous immunoglobulin (IVIG, 1 g/kg per dose, intravenous infusion) was administered on postnatal days 1, 4, and 10. During this period, due to active bleeding or prevention of severe bleeding, random donor platelets were transfused on days 2, 3, and 8 after birth, each time at 10–15 mL/kg. In addition, because anemia met the transfusion threshold, packed red blood cells were transfused on days 5, 6, 8, 12, 19, and 24 after birth, each time at 10–15 mL/kg. Fresh frozen plasma was transfused on days 6 and 7 after birth, each time at 10–15 mL/kg, to correct coagulation abnormalities and hypofibrinogenemia. Albumin was transfused on days 7 and 32 after birth, each time at 10–20 g/L, to correct hypoalbuminemia. However, the platelet count increments were not sustained. Serological tests for anti-human platelet antigen (HPA) and anti-human leukocyte antigen (HLA) class I antibodies were negative. Bone marrow aspiration revealed active proliferation of the granulocytic and erythroid lineages, but dysmegakaryopoiesis (impaired megakaryocytic development). Whole-exome sequencing identified a heterozygous missense variant in the TCF3 gene (c.1676G > A, p.Arg559Gln), which was classified as a “variant of uncertain significance.” To demonstrate the improvement patterns and duration of key laboratory and imaging findings, the dynamic evolution of the main investigations is summarized in Table 1.

**Table 1:** Dynamic evolution of major laboratory and imaging investigations.

Investigation	Time (day of life)	Result	Clinical significance
Platelet count	Day 1	26 × 10⁹/L	Severe thrombocytopenia, primary cause of active bleeding
	Day 14	Began to rise persistently	Initiation of endogenous hematopoietic recovery
	Day 19	>150 × 10⁹/L	Normalized, bleeding risk significantly reduced
Cranial ultrasound	Day 3	Grade II IVH (bilateral subependymal hemorrhage)	Early intracranial hemorrhage, consistent with extreme prematurity
	Day 8	Progressed to grade III IVH with ventricular dilation (maximum width 16 mm)	Hemorrhage progression, associated with severe thrombocytopenia and PDA
	Day 35	Left subependymal hemorrhage 8 × 3 mm, mild bilateral ventriculomegaly	Absorption phase of hemorrhage, ventricular dilation improved
Cranial MRI	Day 34	Grade III IVH with ventricular dilation (old hemorrhage)	Confirmed old hemorrhagic changes, not active bleeding
	Day 49	Absorption phase changes with ventriculomegaly, no parenchymal injury	Significant hemorrhage resolution, favorable neurological prognosis
Cerebrospinal fluid examination	Day 17	Nucleated cells 34 × 10⁶/L, protein 1.91 g/L, glucose 1.17 mmol/L	Attributed to hemorrhage, intracranial infection excluded
	Subsequent	Gradually normalized	Effective response to serial lumbar punctures
Bone marrow aspiration	During hospitalization	Active granulocytic and erythroid hyperplasia, dysmegakaryopoiesis	Confirmed impaired platelet production, consistent with extreme prematurity
Echocardiography	Day 3	PDA diameter 4 mm, left-to-right shunt at great artery level	Hemodynamically significant PDA, increased risk of pulmonary hemorrhage
	Day 27	PDA closed	Effective response to ibuprofen therapy
Platelet antibodies	During hospitalization	Negative for anti-HPA and anti-HLA class I antibodies	Not supportive of alloimmune thrombocytopenia
Infection-related tests	During hospitalization	Blood culture, blood NGS, and CSF NGS all negative	Excluded pathogenic infection
Whole exome sequencing	During hospitalization	No definitive pathogenic variant identified	Not supportive of known hereditary thrombocytopenia

IVH, intraventricular hemorrhage; PDA, patent ductus arteriosus; MRI, magnetic resonance imaging; HPA, human platelet antigen; HLA, human leukocyte antigen; NGS, next-generation sequencing; CSF, cerebrospinal fluid.

### 2.4 Clinical course and discharge status

With comprehensive supportive treatment, the infant's pulmonary and gastrointestinal hemorrhage were controlled. The patent ductus arteriosus (PDA) closed following ibuprofen treatment, sepsis resolved, and respiratory support was gradually weaned down to nasal cannula oxygen. The specific course was as follows: Pulmonary hemorrhage was controlled on day 4 after birth following endotracheal epinephrine administration, hemostatic therapy, and transfusion support. On day 8 after birth, invasive mechanical ventilation was discontinued and switched to non-invasive ventilator support. On day 12 after birth, gastrointestinal bleeding was controlled after fasting, acid suppression, and platelet transfusion, and feeding was resumed. Infectious markers and body temperature normalized, and blood culture and NGS showed no abnormalities; it was comprehensively determined that sepsis had resolved around day 22 after birth. Oral ibuprofen was administered on days 14, 21, and 26 after birth at a dose of 10 mg/kg every 24 h for a total of three courses. Echocardiography on day 27 after birth confirmed that the patent ductus arteriosus (PDA) had closed. On day 33 after birth, non-invasive ventilator support was discontinued and switched to routine nasal cannula oxygen therapy. The platelet count began to rise consistently on day 14 of life and normalized (＞150 × 10⁹/L) by day 19, with no further bleeding episodes thereafter (Figure 2). The amplitude-integrated electroencephalogram (aEEG) tracing improved from moderately abnormal to normal. The patient was discharged on day 42 of life, with a corrected gestational age of 36 weeks and a weight of 2.28 kg. Neurological examination revealed no significant abnormalities, and outpatient follow-up was arranged.

**Figure 2:**
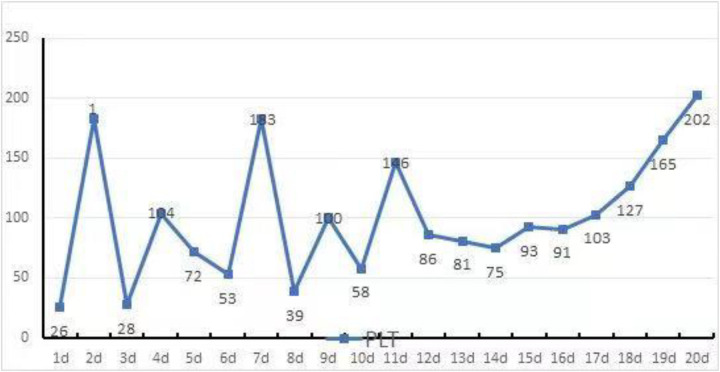
Platelet count trend over time, with a gradual increase beginning on day 14 post-birth and normalization achieved by day 19.

### 2.5 Definitive diagnosis and differential diagnoses

The definitive diagnosis of this case is NITP secondary to maternal ITP, complicated by multisystem complications of extreme prematurity. The specific discharge diagnoses include 23 items: neonatal respiratory distress syndrome, grade III IVH with hydrocephalus, extremely low birth weight, severe thrombocytopenia, neonatal pulmonary hemorrhage, gastrointestinal bleeding, neonatal pneumonia with type I respiratory failure, neonatal sepsis, secondary hyperaldosteronism, hemodynamically significant PDA, neonatal asphyxia, encephalopathy of prematurity, and others.

NITP is an immune-mediated disorder in which maternal ITP-associated antiplatelet IgG antibodies cross the placenta, leading to fetal and neonatal thrombocytopenia. The mother of this case had a 10-year history of ITP, with platelet counts dropping as low as 4 × 10⁹/L during pregnancy, requiring intermittent recombinant human thrombopoietin therapy. The infant presented with severe thrombocytopenia (nadir 26 × 10⁹/L) immediately after birth, which could not be explained by infection or medications, meeting the diagnostic criteria for NITP. Although the infant's platelet antibody test was negative, the maternal ITP background combined with the clinical course is sufficient to establish the diagnosis — negative antibody results are common in neonates, possibly due to insufficient sensitivity of the assay or antibody characteristics (e.g., non-classical antigen specificity or low-affinity antibodies).

Differential diagnoses: The differential diagnosis of neonatal thrombocytopenia is broad. Stanworth et al. noted that it includes potentially life-threatening conditions such as bacterial sepsis, viral infections, and necrotizing enterocolitis (17). The following conditions should be considered in this case:

1. Neonatal alloimmune thrombocytopenia (NAIT): NAIT is an important cause of early-onset thrombocytopenia, accounting for approximately 5% of cases. NAIT is caused by maternal alloantibodies against fetal platelet antigens inherited from the father (e.g., HPA-1a, HPA-5b). The mother's own platelet count is normal, and only the fetus/neonate is affected. In this case, the mother has a clear history of ITP (autoimmune background), not alloimmunity; the infant's platelet antibody test was negative, arguing against NAIT. It has been reported that fetal/neonatal alloimmune thrombocytopenia (FNAIT) affects only fetal platelets and does not affect the maternal platelet count (18); in contrast, the mother in this case had significant thrombocytopenia, consistent with ITP rather than NAIT.
2. Neonatal sepsis-associated thrombocytopenia: The infant was diagnosed with neonatal sepsis and received anti-infective therapy due to prematurity, premature rupture of membranes, and clinical presentation. Sepsis can cause thrombocytopenia through endothelial injury, disseminated intravascular coagulation (DIC), and immune-mediated consumption, and is one of the most common causes of thrombocytopenia in the neonatal intensive care unit (NICU) (17). However, in this case, thrombocytopenia was present at birth (early-onset), whereas sepsis-associated thrombocytopenia typically occurs later (>72 h) (19). Moreover, although infection was controlled after approximately 22 days of anti-infective therapy, the platelet count did not begin to rise until day 14, indicating that the severity and duration of thrombocytopenia cannot be fully explained by sepsis alone. Nevertheless, sepsis may have served as an aggravating factor that increased platelet consumption.
3. Impaired platelet production due to extreme prematurity: The infant was born at only 28 weeks and 3 days of gestation, and bone marrow examination confirmed dysmegakaryopoiesis, consistent with the hematopoietic immaturity of extremely preterm infants. Studies have shown that extremely preterm infants have limited platelet production reserves because of immature hepatic and bone marrow hematopoiesis, which may serve as a baseline contributing factor (19). Such thrombocytopenia is usually mild to moderate (50–100 × 10⁹/L) and typically resolves spontaneously within 10 days. The severe degree (nadir 26 × 10⁹/L) and prolonged duration (>2 weeks) of thrombocytopenia in this case cannot be explained by this mechanism alone.
4. Hereditary thrombocytopenia: Whole exome sequencing did not identify any definitive pathogenic variant, which does not support a diagnosis of known hereditary thrombocytopenias (e.g., MYH9-related disorders, Bernard-Soulier syndrome, Wiskott-Aldrich syndrome). Such disorders usually present with a family history or specific clinical features (e.g., giant platelets, eczema, immune deficiency), none of which were present in this case.
5. Other causes of early-onset thrombocytopenia: These include chronic fetal hypoxia related to placental insufficiency [e.g., maternal preeclampsia, diabetes, intrauterine growth restriction (IUGR)], perinatal asphyxia, and congenital viral infections (19). The infant had a history of IUGR, which could explain mild thrombocytopenia but not the full picture of severe thrombocytopenia and multisystem bleeding. TORCH (toxoplasmosis, rubella, cytomegalovirus, herpes simplex, and others) screening was unremarkable, ruling out congenital infection.

Relationship between multisystem signs and the diagnosis: All of the multisystem signs in this case (grade III IVH, pulmonary hemorrhage, gastrointestinal bleeding, severe thrombocytopenia, sepsis, PDA, respiratory failure, etc.) can be attributed to a comprehensive clinical scenario with immune-mediated thrombocytopenia secondary to maternal ITP as the core, superimposed on the inherent vulnerabilities and complications of extreme prematurity. Specifically, maternal ITP led to severe thrombocytopenia from birth, directly predisposing to bleeding. The immature hemostatic system, fragile cerebral vessels, and mucosal barriers of the extremely preterm infant provided the substrate for bleeding. The high pulmonary blood flow state caused by PDA increased pulmonary vascular pressure and the risk of pulmonary hemorrhage. Sepsis-induced endothelial injury and coagulopathy further exacerbated the bleeding tendency. The superposition of these multiple factors resulted in extensive and severe multisystem bleeding. Therefore, this case is not a single disease explaining all manifestations, but rather a complex clinical syndrome with immune-mediated thrombocytopenia at its core, intertwined with multiple contributing factors.

## 3 Discussion

This case report describes a very preterm infant born to a mother with ITP who presented with severe thrombocytopenia and multisystem (intracranial, pulmonary, and gastrointestinal) hemorrhage in the early postnatal period. While IVH and pulmonary hemorrhage are not uncommon in very preterm infants and are often associated with factors such as PDA, sepsis, and respiratory distress, the co-existence in this infant of severe, persistent thrombocytopenia that responded suboptimally to initial IVIG and platelet transfusions strongly suggests that the immune mechanism related to maternal ITP was a key factor exacerbating the bleeding risk and complicating the clinical management. The following discussion integrates insights from this case.

### 3.1 Complexity and multifactorial pathogenesis of NITP in very preterm infants

The clinical presentation of NITP in very preterm infants is often the result of the superposition of multiple factors. This case illustrates a triple interaction: (1) Maternal antibody-mediated immune destruction: The mother's long-standing history of ITP and severe prenatal thrombocytopenia suggest the presence of high levels of antiplatelet antibodies. These IgG antibodies cross the placenta, leading to fetal platelet destruction. Although the infant's antibody tests were negative, this is common in neonates and may be related to insufficient sensitivity of the assay or antibody characteristics; thus, an immune mechanism cannot be ruled out (20–24). (2) Gestational age-related limitations in platelet production: Bone marrow examination confirmed dysmegakaryopoiesis, consistent with the immaturity of the hematopoietic system in very preterm infants, resulting in insufficient platelet production reserve (25, 26). This made it more difficult for the infant to maintain adequate platelet counts on top of the immune destruction. (3) Potential immune dysregulation: delayed response to multiple courses of IVIG suggest the possibility of individualized dysfunction in immune handling or clearance (e.g., differences in FcRn-mediated metabolism) (10–12, 15), although the exact mechanism requires further investigation.

From the perspective of differential diagnosis, other common causes of early-onset thrombocytopenia need to be excluded in this case. The differential diagnosis of neonatal thrombocytopenia is broad, including bacterial sepsis, viral infections, and necrotizing enterocolitis (17). Stanworth et al. emphasized that distinguishing these etiologies from hereditary thrombocytopenias and fetal/neonatal alloimmune thrombocytopenia (FNAIT) is crucial for accurately assessing serious adverse events such as intracranial hemorrhage and for selecting treatments such as platelet transfusion (17). In this case, although neonatal sepsis was present, thrombocytopenia was already evident at birth (early-onset), whereas sepsis-associated thrombocytopenia typically occurs later (>72 h) (19); FNAIT was excluded because the mother had a normal platelet count and the infant tested negative for platelet antibodies (18); whole exome sequencing did not identify any definitive pathogenic variant, ruling out known hereditary thrombocytopenias. Therefore, immune-mediated thrombocytopenia secondary to maternal ITP was established as the core etiology of thrombocytopenia in this case.

### 3.2 Risk stratification and management implications in multisystem hemorrhage

The hemorrhage events in this infant occurred against the inherent vulnerability of extreme prematurity—characterized by an immature hemostatic system, fragile cerebrovasculature, and mucosal barriers—and were further compounded by a patent ductus arteriosus (PDA, increasing pulmonary blood flow and hemorrhage risk), sepsis (causing endothelial injury and coagulopathy), and most critically, severe immune-mediated thrombocytopenia. This “multiple-hit” model explains the extensiveness and severity of the bleeding. This highlights for clinicians that for very preterm infants with a maternal ITP background, risk assessment and management of bleeding must be multidimensional and dynamic, requiring simultaneous attention to platelet counts, coexisting conditions (e.g., PDA, infection), and the functional status of organs.

### 3.3 Re-evaluation and optimization of treatment strategies

The therapeutic response in this case posed a challenge to conventional management protocols. The limited efficacy of the standard IVIG course suggests that in very preterm infants, individualized dosing or administration strategies may be necessary, taking into account their FcRn-mediated IgG metabolism characteristics (10–12). The transient effect of multiple platelet transfusions, coupled with the limited benefit of indiscriminately pursuing HLA-matched platelets once an autoimmune background is confirmed (5, 27), supports the notion that in ITP-associated NITP, transfusions should primarily be reserved for controlling active bleeding rather than merely aiming to increase platelet counts. This case shares typical similarities with the “unstable patients with platelet counts <50 × 10⁹/L” in the NICU and the “early-onset thrombocytopenia of unknown cause” described in Table 1 of the report by Murray et al. (19) — both characterized by preterm birth, early onset, marked thrombocytopenia with multisystem bleeding, and a specific dynamic response pattern to platelet transfusions. Murray et al. pointed out that in such patients, platelet counts may reach 50–100 × 10⁹/L one hour after transfusion but often fall to <50 × 10⁹/L within 24 h, reflecting moderate platelet consumption (presumably mediated by maternal antiplatelet antibodies) and impaired platelet production in preterm infants (associated with dysmegakaryopoiesis), and predicting a favorable response to platelet transfusion therapy. In the present case, two episodes were also observed in which platelet counts rose to the range of 50–100 × 10⁹/L one hour after transfusion but fell to <50 × 10⁹/L within 24 h, consistent with the description in that report and supporting the mechanism of immune destruction combined with impaired production. According to the recommendations of that report (see its Figure 1), platelet counts should be repeated every 6–12 h after platelet transfusion in such patients; if the post-transfusion count falls below 50 × 10⁹/L, further transfusion should be considered. In the present case, a similar strategy was adopted during the active phase of pulmonary and gastrointestinal bleeding, and multiple transfusions controlled active bleeding. The report also noted that more frequent platelet transfusions may be sufficient to control bleeding without necessarily requiring other specific therapies (e.g., high-dose IVIG or second-line agents), which echoes the actual course of this case — poor early response despite multiple courses of IVIG, and stabilization of platelet levels relying on transfusions. However, due to the presence of multiple systemic complications of extreme prematurity and potential genetic factors, this case ultimately required a combination of IVIG and comprehensive supportive care to achieve platelet recovery, suggesting that the protocol derived from the literature should be adjusted according to individual risks and comorbidities.

This case demonstrates a staged, multidisciplinary collaborative comprehensive treatment strategy: an escalation approach was adopted for anti-infective therapy to ensure coverage of potential pathogens; in hemorrhage management, platelet, plasma, and albumin transfusions were administered as needed along with pharmacologic hemostatics; respiratory support was gradually de-escalated to reduce the risk of BPD; timely ibuprofen was used for PDA management; and in thrombocytopenia management, IVIG was combined with frequent platelet transfusions, with platelet counts rechecked every 6–12 h after transfusion and repeat transfusions given promptly as recommended by Murray et al. Implementation of the above regimen achieved positive outcomes including platelet recovery, hemorrhage control, infection resolution, and stabilization of multiple organ functions, providing a reference for the clinical management of similar cases of extremely preterm infants with NITP complicated by multisystem involvement.

### 3.4 Future research directions

This case underscores the importance of integrating clinical practice with basic research. Establishing a multicenter registry system dedicated to NITP in very preterm infants, systematically collecting clinical, immunological, and genetic data, is crucial for identifying high-risk phenotypes, elucidating pathogenic mechanisms, and evaluating treatment efficacy. Future research should focus on integrating this information to develop risk stratification tools and mechanism-guided, individualized management pathways, thereby improving outcomes for this vulnerable population.

## 4 Conclusion

We report a case of a very preterm male infant born at 28 weeks and 3 days of gestation to a mother with ITP, who experienced severe early-onset thrombocytopenia and multisystem hemorrhage. The definitive diagnosis of this case was NITP secondary to maternal ITP, complicated by multisystem complications of extreme prematurity. The infant's clinical course exemplifies the intricate interplay and combined effect of common complications in very preterm infants, such as PDA and sepsis, with immune-mediated thrombocytopenia secondary to maternal ITP. Despite the presence of multiple confounding factors, the severity and distinctive therapeutic response of the thrombocytopenia underscore the central role of NITP. This case supports a multifactorial pathogenesis for NITP in very preterm infants, involving maternal antibody-mediated destruction, impaired fetal platelet production, and potential individual immune-genetic predispositions. All multisystem signs can be attributed to a comprehensive clinical syndrome with immune-mediated thrombocytopenia as the core, superimposed on the inherent vulnerability and complications of extreme prematurity. This highlights the necessity of a comprehensive management strategy for such infants, which should actively assess and provide individualized interventions for the immune thrombocytopenia while concurrently addressing general problems associated with prematurity. Future research must integrate clinical, immunological, and genomic data to advance precision medicine for NITP in very preterm infants.

## Data Availability

The raw data supporting the conclusions of this article will be made available by the authors, without undue reservation.
